# Perceptions and Experiences of Older Persons in Two Types of Institution in France: Foster Care Family Institution and Medico-Social One

**DOI:** 10.3389/fpubh.2021.684776

**Published:** 2021-09-06

**Authors:** Rita Chammem, Serge Domi, Anne Marie Schott

**Affiliations:** ^1^University Claude Bernard Lyon 1, Lyon, France; ^2^Department of Sociology, Crifondo, Martinique; ^3^Hospices Civils de Lyon, Lyon, France

**Keywords:** aging, foster care family, older people, inclusive housing, institutionalization

## Abstract

**Background:** The rapid increase in the number of older people with loss of autonomy and requiring human support, medical services, and adapted accommodation is a challenge in many countries. Beside institutions for older people, foster care families may offer an interesting innovative and affordable alternative that should to be evaluated. As the place of living has a major impact on the quality of life, our objective was to compare the perceptions and experience of older adults depending on the type of accommodation in which they live in.

**Methods:** This study was based on a mixed method approach, with a quantitative assessment and a qualitative study with semi-structured interviews conducted with older people living in two different types of institutions: medico-social institution and foster care families. We explored the perceptions and the quality of life of the participants.

**Results:** Institutionalization, chosen or not, can represent a trauma for older people as it disrupts their personal habits. Participants described medico-social institutions as an institutional framework supported by a strict professional team and rules, as impersonal structures (i.e., somewhat neutral), and as offering a very present, prepared, and respected reception protocol on the first day of arrival, which remained marked in memories. On the other hand, foster care families were considered as more spontaneous and family-oriented structures relying on internal and negotiable rules, offering personalized and close support in the face of loss of autonomy, but in which the reception protocol on the first day of arrival was almost absent.

**Conclusions:** Foster care families and medico-social institutions presented different advantages and disadvantages from the point of view of the participants. This can help health policy makers to rethink the way seniors are housed by taking into consideration their perception and quality of life.

## Introduction

Population aging is a global phenomenon that affects both developed countries, such as Japan, and emerging countries, such as China (1). The European population is also aging and in 2018, 33% of European people were aged 55 years and older (2). A similar trend is observed in France, although it differs between metropolitan France and French overseas territories, where aging is particularly rapid, especially in Guadeloupe and Martinique. Martinique used to be one of the youngest French territory, but its demographic status has rapidly changed with a significant increase in the number of older adults to the point that by 2030, 40% of its population will be aged 60 years and older and it will be one of the three oldest French regions (3). This rapid demographic transformation is also specified and explained by the combination of five simultaneous demographic phenomena (4).

In France, the supporting institutional offer is rather binary: either staying at home with services or moving to an accommodation structure for older adults like a medico-social institution or a foster care family, and these decisions do not depend only on individual choices. However, while keeping seniors at home is a widely-desired option today as 81% of French people wish to die at home (5), frailty and the institutionalized end-of-life often becomes unavoidable. Thus, the care of older people should combine at the same time familial solidarity and institutional solidarity for the well-being of older individuals (6) and the relief of the caregiver.

Due to the absence of familial caregiver or to their difficulties (7), a large number of older people do not have the possibility to stay safely at home with an appropriate support. Therefore, informal caregivers are necessary to support the de-institutionalization process (8). As the population ages, caregivers are themselves becoming older and developing disabilities and chronic diseases (9). Moreover, the case of paid caregivers of their parents represents a special situation of this work of “care.” This mode of support is not without problem, such as the exhaustion of the worker—as he/she often combines a paid work of care and a free activity of caregiving (10), the tensions between siblings, the lack of qualification of the caregiver to cope with certain pathologies of old age, particularly Alzheimer's disease and Alzheimer-related diseases. Moreover, the work of “taking care” and “caring for” is inconspicuous and poorly paid as it is poorly recognized.

Indeed, the existence of end-of-life institutions remains largely unknown by older people and their family (11) and they seem to struggle developing (12, 13), despite their being available since the days of the French Revolution (14). In 1989 (15), a law was promulgated to create approved foster care family institutions in order to support older people who are losing their autonomy and adults with disabilities (16).

Foster care families welcome older individuals or/and adults with disabilities in the household of approved individuals who do not belong to their family (up to four degrees inclusive) against a remuneration determined regarding the minimum legal salary in France.

Welcoming individuals (hosts) must be approved (an approval issued by the Chairman of the Departmental Council for a period of 5 years, renewable if the required conditions are still validated). They are also controlled every month by a structure mandated by the General Council on which they depend to ensure a good welcome.

In Martinique, the rather unexpected demographic situation is worsened by economic and social factors. Economically, the situation is delicate since this aging is associated with a situation of economic vulnerability for many older people (4). Although the geographic proximity (small size of the island) has been shown to be a factor favoring exchanges of services and informal care between generations and encourage the solidarity, the geographical and socio- economic characteristics of the island drives young people to leave the island and change the size and notion of family (17).

In an attempt to adapt to the demographic changes, this outermost region has been equipped with a number of services facilities and innovative devices, however their capacities (49 places per 1,000 inhabitants aged 75 years and older) remain lower than in metropolitan France (123 places per 1,000 inhabitants aged 75 years and older)^1^.

The foster care family activity has been set up by the local authority of Martinique since December 1990^2^. Since then, the local authorities (CTM) have mandated an association to work for the development and the proper functioning of the system. In 2015, there were 74 accredited families, able to welcome a total of 177 people.

Housing is an element of the basic context of living conditions and crosses a wide range of topics such as health, intergenerational links, network, mobility, and social participation that have a real impact on the independence of older people, that is why it has become a major social and public health issue (18).

The entry in an institution is most often experienced by older patients and their entourage as a brutal upheaval (19–21) and as a turning point in their life. However, little data are available regarding the way entry into a foster care family is experienced. Intuitively, one might think foster care families would be appreciated as they represent, in a way, an extension of the family. They may appear as an intermediate modality between staying at home and being placed in a medico-social institution, thus prolonging the at-home experience before the often unavoidable medical institutional placement.

**Figure 1:**
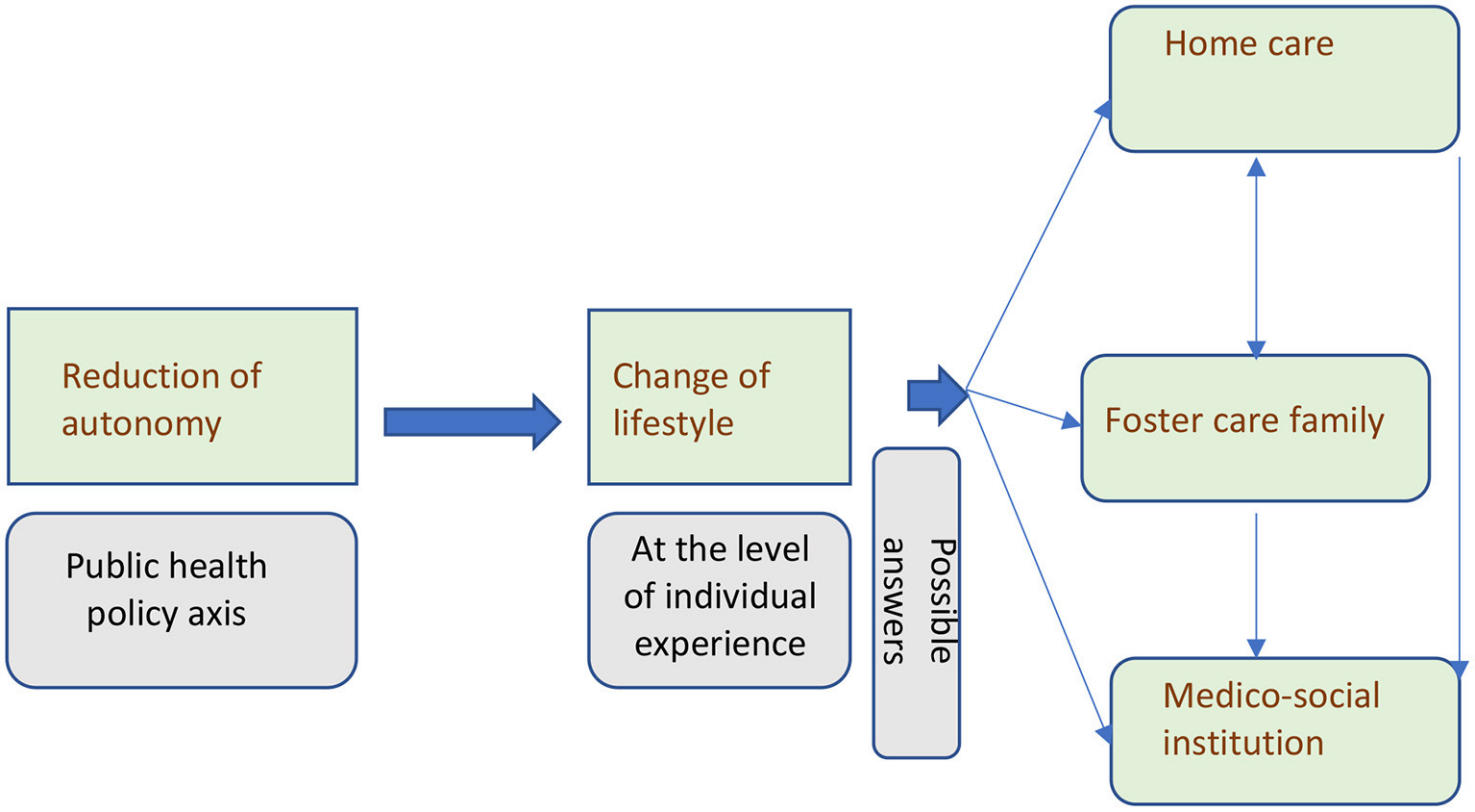
Loss of autonomy and change of lifestyle.

Figure 1 represents the hypothesis that foster care family may be considered as an intermediary offer between two options: home support and medico-social institutions. It offers an alternative in-between formula, which makes it possible to consider moving from one situation to another, to improve autonomy, to check the possibility of a return to home, and to expand the range of accommodation solutions.

In an overseas field, we studied the national system of foster care family that comes as a substitute for the family and presents itself as a form of intermediate accommodation for older persons. This intermediary offer between home stay and institutionalization could respond to requests for long-term or temporary support, especially during the holidays, after a hospitalization, to provide respite for the informal caregivers, in preparation of a medico-social institutional placement, or, why not, to manage situations of health crises and “shortness of breath” of medico-social institutions.

The present study should therefore make it possible to identify innovative ways of understanding the needs and the preferences of the older adults and widen their autonomy.

How to use these experiences to adapt the offer of institutions to an older, dependent population?

## Methods

To understand what the foster care families, relatively unknown in metropolitan France, correspond to, we performed an analysis comparing the experience of older individuals in foster care families and in medico-social institutions. The aim was to better assess from the point of view of older people whether this foster care family system was actually experienced as an intermediary solution between at-home living and placement in a medico-social institution, and whether it could prolong in a certain way the at-home experience, even in the absence of actual family members.

A qualitative approach was used in the present study. We conducted a qualitative comparative analysis of the quality of life, well-being, and experience of older persons living in foster care families or in medico-social institutions, such as medico-social institution; and to provide a more in-depth individual analysis.

### Qualitative Study

This study took place from March; 2018 to March, 2019 and was based on an ethnographic approach consisting of a qualitative survey based on interviews following the COnsolidated criteria for Reporting Qualitative research (COREQ) (22).

Non-probability sampling (23) was undertaken until saturation. This is achieved when additional information is no longer available to enrich the latest data collected and analyzed (24).

Table 1 shows criteria for recruitment.

**Table 1:** Participant recruitment criteria.

**Inclusion criteria**	**Non-inclusion criteria for**
- Age: 65 years and over, - Place of living: medico-social institution or in foster care family, - Ethics: informed consent obtained after being informed of the study both in writing and orally.	- Physical health: inability to participate because of illness, hospitalization, or incapacity on the day of interview, - Mental health: Mini Mental Status Examination (MMS) score <25 (25).

### Method of Sampling

From our professional networks, samples were selected through a key contact. Participants were selected in the two groups (medico-social institution and foster care families) with attention to socio-demographic parameters (sex, age, marital status, children, location, and municipality of origin).

#### Concerning Foster Care Family

The association AMDOR (*Association Martiniquaise pour la Promotion et l'Insertion de l'Age d'Or*) manages foster care families, is in possession of all the information concerning this system, and provided the list of foster care families. We then carried out a stratified random draw to obtain a fairly varied sample for qualitative interviews.

#### Concerning the Medico-Social Institution

Medico-social institutions were identified from the list provided by the local health agency (ARS: *Agence Régionale de Santé*) and the CTM, based on the following criteria: seniority on the island, reception capacity, geographical situation, Weighted Average of medium dependency level (GMP) in comparison with the average and median GMP, as well as the services offered.

The list of participants was provided for each medico-social institution by the occupational therapist and psychologist, to whom the criteria of inclusion and non-inclusion were communicated.

### Data Collection

Individual semi-direct face-to-face research interviews were conducted with the participants of both groups at their place of life. The interviews followed a topic guide (Table 2) that has been developed during a previous phase by a panel composed of researchers in public health and human sciences and geriatricians, and tested with several older people until saturation (Appendix 1).

**Table 2:** Semi-structured interview guide.

Explored domains	1. Perception of change
	2. Feeling in the institution
	3. What do you like and dislike here?
	4. Perception of quality of life
	5. Perception of autonomy
	6. Knowledge of the institutional offer
	7. Evaluation of the institutional offer
	8. Is there anything else you would like to tell us?

This topic guide allowed to ask participants about their experience in both types of accommodation, their expectations, their habits, their relationship with others, and their experiences of the support provided in each of these modes of accommodation.

Our approach was framed in an inductive way allowing to listen and to let new themes emerge. It also had the advantage of emphasizing the perspectives of the participant (23).

These interviews were conducted individually, recorded on a Dictaphone, and then fully transcribed in order to conduct the most accurate content analysis possible.

### Data Analysis

The use of theory to guide the analysis was an iterative choice. Review of the literature had been performed prior to analysis while no specific theoretical framework had been chosen. A first step of inductive analysis was conducted independently by two authors (RC and SD) from different disciplines and teams. Firstly, anonymized manuscripts were read in their entirety to get a holistic view of the data set and get familiar with the topic. In a second step, these two same authors worked together to reach a consensus regarding the analysis of the contents.

The body of data collected was analyzed regarding the original objective of the study and using conventional qualitative thematic analysis (26). Interviews were analyzed line-by-line and the data set was coded for content.

**Figure 2:**
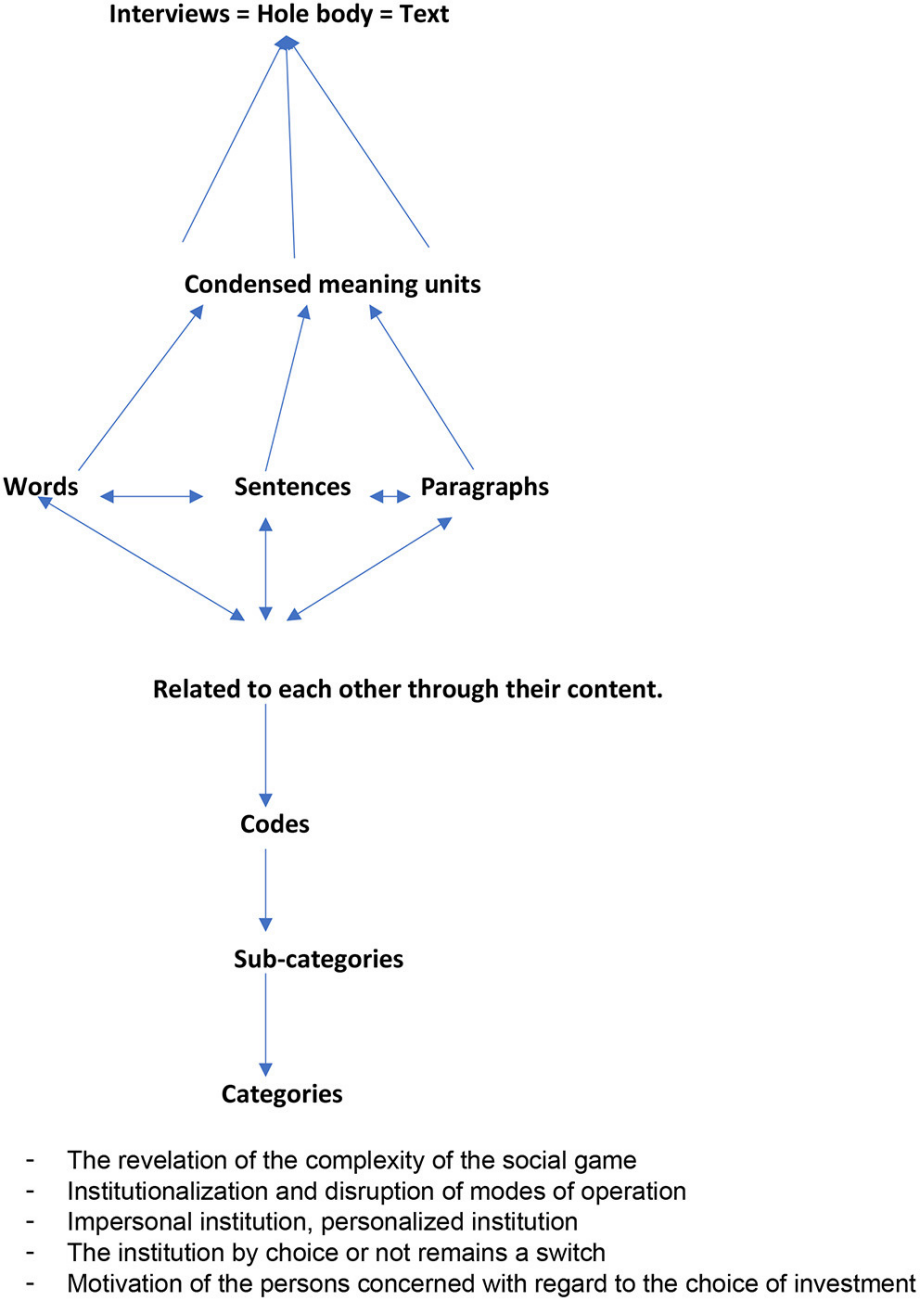
Thematic analysis; from interviews to categories.

A thematic content identified the lexicon used by participants to describe their experience. The categorization of these codes into sets of thematic categories allowed to develop a thematic map (Figure 2) that represents the themes, sub-themes, and primary codes. It was further refined during discussions with inputs regarding interpretation from the research team during the drafting of the manuscript.

### Ethics

This study was in accordance with the applicable legislation and regulations. The ethics Institutional Review Board of the INSERM (*Institut national de la santé de la recherche médicale*, French institute of medical research), with the flowing identification: IRB00003888, IORG0003254, FWA00005831, approved this study. Written permission was addressed under the number IRB00003888, IORG0003254, FWA00005831. The study was also compliant to the CNIL (*Commission nationale de l'informatique et des libertés*, French commission on data protection) MR-003 methodology.

Participation was voluntary. Participants gave their oral consent for their participation in the study, which was recorded. The participant informed consent included the publication of anonymized responses.

Identity and medical information of the participants were not collected. The anonymity and confidentiality of the data collected and analyzed were assured to the participants. All involved in the conduct of the study or the analysis of results were held to the strictest professional secrecy.

Participants were informed by a written informative note describing the purpose, date, place, and course of the study. The elements of this brief note gave the individuals an opportunity to reflect on their participation and take the time to make their decision. After consent, the participant remained free to leave the study at any time without providing an explanation. Participants were informed that, at the end of the study, the results of the research could be communicated upon request to the investigator of the study.

## Results

### The Foster Care Family, an Example From the Martinique Region

The number of approved foster care families and the number of active foster care families changes every year (minimum of 67 approved families in 2007 and maximum of 80 approved families in 2006) due to the unbalance between the number of new approvals and the number of removal of families from the system. Besides approved families, so-called “wild” foster care families also exist. These families host at home and for a fee (minimum 2000€/senior/month) one or several older person (people) without being approved, in other words, they illegally engage in the hosting activity. Some of these illegal hosts managed to obtain their approval after a few years of “illegal” practice, others refused to apply or had their approval refused. Conversely, some initially approved hosts subsequently lost their approval following a sanction for serious reasons, but continue to carry out the activity illegally. We do not have quantitative data to describe this phenomenon. Nevertheless, there seems to be more un-approved than approved foster care families.

The significant amount of applications from seniors and those around them, the long waiting list for admission into residential institutions, and the lack of institutional space may explain the existence of these un-approved foster care families. Unfortunately, it was impossible to obtain data regarding the number of people on waiting lists or to have the opportunity to talk to them.

Since 2014, 33 (45%) approved families are in the central areas, 28 (38%) in the south, and 12 (17%) in the northern area. Although this may suggest a higher concentration of older people in the central and south urban area, demographic data indicate the northern zone population is the oldest (44% of the population is aged 60 years and older). This central concentration of foster care families may result from a choice of the relatives of older people as they represent an active population who lives and/or works mainly in the central areas of the island.

The activity of foster caring is mainly carried out by women (86%) although recently (since 2010) more men are asking for habilitation. This inequality between sexes is observed in most occupations of personal assistance, which are often considered as precarious and requiring a low level of skills, and in occupations from the social sector. The age of foster care givers ranged from 36 to 70 years, and 71% of women were aged between 46 and 60 years old.

Maritally, it turns out that 60% of hospitable couples live together and 40% are not. It may be thought that hosts who live in a couple benefit from the support of their spouse and offer the hosted persons an environment similar to a traditional family.

However, it remains to be verified that the fact of not living as a couple means that people live alone. In the present study, although hosts were not in a relationship, they often lived with family members or with a friend, who supported them and helped them to carry out their activity. Also, the long daily visits of relatives reflect the strong existence of the notion of extended family.

Seventy-two percentage of the hosts owned their accommodation and 3% lived in apartments, the others (1%) occupy a family accommodation. Accommodation were usually poorly adapted and poorly equipped for seniors.

The majority of older welcomed people received financial assistance from the CTM.

The number of departures (including deaths) varies from year to year. For instance, there was 32 departures in 2014 to another type of accommodation, including the return at home.

### The Medico-Social Institution and the Foster Care Family Institution: Supply and Demand From the Perspective of Older People

A total of 24 interviews were conducted with 12 older people living in foster care families and 12 older people living in medico-social institution. The mean (range) age was 81 (65–94) years (Table 3).

**Table 3:** Participant characteristics.

**Characteristics**	**Number**		
	**Foster care families**	**Medico-social institutions**	
Sex	Male	7	4
	Female	5	8
Age	65–75 years	6	2
	76–85 years	3	3
	Over 85 years	4	7
Child(ren)	Yes	9	10
	No	3	2
Health status	Diabetes	4	2
	Cardiovascular disease	2	4
	Stoke	1	2
	Depression	2	3
	Deafness	1	5
	Blindness	1	1
	Physical handicaps	2	1

The interviews lasted 45 min on average. They revealed a number of points concerning the functioning, reception/welcoming, choice, advantages and disadvantages of the two types of institutions studied from the point of view of institutionalized persons.

#### The Revelation of the Complexity of the Social Game

Older people hosted in foster care families expressed a certain sense of freedom in decision making (“*I am free here, I do as I am pleased*,” “*I go out when I want*,” “*every morning, I go for my morning ride*”), and usually their host manages the daily chores: “*the host has his/her job*.” On the other hand, the rules in medico-social institutions were described as strict and the employees as “*authoritarians,” “who like to command*,” “*more or less kind*,” and with an “*an attitude that you don't like*.”

The interviews also highlighted that the host in foster care families is a person with whom negotiation remains somehow possible. Conversely, in medico-social institutions, negotiation was found as not even possible for “simple habits” of daily life as older people are rarely in direct contact with the actual decision makers and as rules have to be strictly followed for organization purpose. This applied to meals (“*They don't let us put salt on the table*,” “*I can't stay at my room for lunch*”), the time for bath, the participation to the proposed activities (“*They don't want to understand when you say you don't want to go out*”), etc. Staff members make decision for residents, while they felt that they are “*old enough and can decide*” (“*When I say NO, you have to understand me*” and “*I don't like it when I'm not allowed to go out*”). Some residents do sneak around these rules: “*So, I stay alone in my room*” and “*I sprinkle my dishes with the salt I bought myself*.”

#### Privacy and Disruption of Personal Habits

The notion of privacy was addressed by the two groups of respondents.

In foster care families, welcomed people (guests) have to share all accommodations, sometimes even their bedroom. Sharing the same place of life with other people bothered them (“*I am used to living alone*”) especially since they do not live at home (“*this is the first time I've been to people's homes*”). Guests also mentioned: “*it's my privacy*” being impacted as “*shared the bathroom*.”

In medico-social institutions, people have their own bedroom and bathroom, and the issue was approached differently: “*I have my own space*” but “*I can tell you everything*” because “*we hear everything here*,” “*we have to be careful*.” Even if they do have their own personal space, residents still have to live in community with other people: “*you have to learn to accept each person with his or her character, his or her defect*” even “*people you can't get along with but have to endure*” and with whom “*it's not easy*,” “*You have to know how to manage living in community*,” “*it's not easy to accept, you have to know how to do the work*.”

The presence of people with little autonomy or/and disabilities in medico-social institutions reflects a negative image of finitude, to which residents were not indifferent: “*a lot of people suffer from dependency here, perhaps I'll be like them also tomorrow*,” “*I used to go out with the neighbors on my institution floor, we were friends… But they are no longer there, they are dead… So, I too will follow*.”

For both modes, privacy referred to the question of the intimate and the collective, even if it was defined differently. For participants living in foster care families, privacy mostly referred to the living space because they had to share it with others, and for participants in medico-social institutions, privacy referred mostly to freedom of expression because of the fear of being heard by others (residents or staff) when expressing a dissatisfaction.

For both groups, a trauma due to the changes related to the temporal organization of the days was identified: “*It is at specific times*” [that we must do the tasks of daily living], “*wake up*…” “… *sleep*…” “…*nap*…” “…*eat*….”

Interestingly, this was a bigger trauma for the group of people living in foster care families, as if, in a way, they did not accept to be “*like at home*” but with constraints. They asked for more freedom, especially regarding the time of awakening (“*I don't like when the nurse comes early and everyone is still sleeping”*) and time of meals (“*We eat at 5 p.m. here because the hosts goes to bed early, for me it is too early*”).

Nonetheless, participants from both groups made a similar emphasis on the morning trauma. In foster care families, some guests require some exterior interventions and must respect the schedule of the professional: “*I wake up to be showered by the nurse*” [and then] “*I sit and wait*.” Such waiting moments seem experienced as if life was collapsing. But also: “Before coming here, *I could get up when I want, but here, there is a time to wake up*….” Residents from medico-social institutions evoked that: “*professionals come to their rooms early in the morning without knocking*…” [they] “…*take the cover off without asking me*…” [and] “… *I'm not awake yet*…” [but] “… *I realize that the professionals are bathing me*…” [and they] “… *talk very loudly to each other without wondering if it would bother me…*.”

#### Personal vs. Impersonal Care

Medico-social institutions is an impersonal structure, which is somehow neutral, it belongs at the same time to no one and to everyone. Additionally, they are composed of a large number of residents with disabilities who require a lot of time from professionals, the latter have therefore very little time to provide personalized care: “*it's that sometimes when you feel bad, you ring, you wait for 3/4 of an hour before someone comes, it happened to me once, twice*”; this introduces the lack of availability of staff members: “*I am starting to weaken and the institution is under staffed*,” but this also evokes the lack of compassion and responsiveness of professionals: “*I'm afraid of falling in the shower alone, I wish there was someone*.”

Foster care families on the other hand offer personalized and close support in the face of loss of autonomy. The number of guests was most of the time limited and they noted that “*there are not many people as in retirement homes, it's just three people here*,” [the host] “*takes care of you more*” and “*there is always someone who looks after me*….”

The reception protocol on the first day of arrival is very present, prepared, and respected, and remains marked in people memories in traditional institutions, but is almost absent in foster care families. From this perspective, the traditional institution seems to pay more attention to the hosting protocol, it gives a formal welcome to its residents and helps them settle in their new place of life, which was much appreciated: “*I found warmth*,” “*I found really nice people*.” People take ownership of the place more quickly: “*my second home*,” “*as if I were at home*.” Adaptation requires a little more time in foster care families, also for the hosts who need acceptance and knowledge of the guests in order to really open the doors of his/her house with confidence. This is a rather unusual situation for both the hosts and the guests, but when their personality match the guest', they become in most cases a family member. In such cases, participants mentioned a successful integration and created links: “*it's very family-like with the foster family, the children come by, the grandchildren*….”

Regardless of the institution, many participants remembered the day (“*on a Monday*,” “*I arrived on a Thursday*”), the date (“*June 4th*”), and the time (“*it was about 9:00 am*”) of their arrival. This confirms that the experience is remarkable, it that remains etched in the memory. Others preferred not to talk about it and said: “*I don't know, it's far*…,” even if they arrived only 6 months or 1 year earlier.

#### The Institutionalization—Chosen or Not—Can Be a Trauma

A variety of reasons lead to the choice of leaving home and getting settled in an institution, they are essentially related to the isolation that makes living at home dangerous for the older people and degrades their autonomy. Few of these choices are personal, thoughtful, and deliberate, and in the majority of cases, participants did not complain and displayed themselves in a state of passive acceptance.

They expressed this choice was made by their relatives (“… *parents*…,” “… *neighbors*…,” “… *social service*…,” “… *sisters*…,” “… nephews…”) and that they were subsequently involved in the choice of either foster care family or medico-social placement.

The foster care family was often perceived as a kind of solution in case of emergency (“… *following hospitalization*…,” “… *My house collapsed*….”).

The hosts also make a choice regarding guests, as they do not allow anyone to enter their family home (moral, religious, or other criteria).

The change associated with the installation in foster care families was less traumatic: “*I was happy* [when I arrived in the foster care family], even though “*I ate in my room the first few days*.” They did not appear as a final step (“*I don't know about tomorrow*…,” “*I asked for help to get my own home*,” “*I don't want to go to asylums*”) but rather as a temporary state, even if it eventually lasts over time (“*I don't know what to do*,” “*people are at home here*,” “*if my sister comes back here, I go home*,” “*I don't want to be buried here, all the ancestors are there, I want to move before I die*…”).

Perhaps, what makes it more bearable is in a way the continuity of the family dimension. However, participants complained about the lack of distractions (“*lack of distractions*,” “*Outside of school holidays, there is no one to play chess with me*,” “*there is not much to do*”) and external social isolation (“*I want to meet people and make new acquaintances*”), and they suffered from a lack of mobility (“*I want to go to the library*,” “*I would like to go out but the bus is far away and I am afraid alone*”).

Conversely, the change (emotional, environmental) associated with the installation in medico-social institution was perceived as strong and brutal (“… *a fear*…,” “… *hard*…,” “*… at first, very hard*…,” “*I didn't take it very well because I know these places*”) but without nostalgia (“*the years that passed will not coming back*,” “*ah it's already pass*ed”). People were no longer seeking to return to the past of their former home or to create a new future, even if they were aware that they “*come to die here*.” The animations were considered unsatisfactory by the majority of residents despite their diversification and frequency proposed by medico-social institutions: “*before there were more trips*,” “*When I came here, we were fine, we went out, we went to the beach, we were sent to Perrine's farm for lunch and all that was good old times*.” Medico-social institution residents also reported a problem: “*you have to pass the time, to pass the time,” “you have to accept, let go, let go, you have to let go*.”

The residents themselves with this expression of boredom drew a situation of neglect and weariness despite the wide range of leisure put with care at their disposal.

This apparent boredom seemed different from that experienced by the foster families' guests who were looking for activities, a project of ordinary life, continuity, and encounters: “*meet with people of my generation*,” “so, *that I'm a little bit connected with people of my generation*.” Household chores become somehow a distraction, “*the host lets us help him, it keeps me busy*,” “*I do the dishes, I clear the table*.”

#### Knowledge of Participants Regarding the Different Systems Available

The majority of people living in traditional institution expressed a lack of knowledge regarding the existence of foster care families and other existing systems: “*I don't know any other system*,” “*I don't know the family home [foster care family]*,” “*I have not been told what the family home [foster care family] is*.” This lack of knowledge was associated with a negative idea of the foster care family system: “*foster homes are for people who do not have families*,” “*for me, a foster home is a house in the countryside*” (for them, it means remote and isolated areas), “*in a foster home, you will die like that, anonymously*.”

They defended their choice, stating they preferred to be in a medico-social institution than to be kept at home in solitude, and advised other seniors by trying to reassure them: “*I tell people who are afraid of the medico-social institution that they are wrong because living alone in a home like that is not good*.”

People who live in foster care family were afraid of ending up in medico-social institutions and called them “*asylum*.” They did not want “*to be sent to asylum*.”

The presentation of the foster care family system is done in an informal way by the hosts, by enrolling in networks of charity (“*hosts visit the hospital*,” “*open their homes to visits*” to make their existence known and attract “clients”), but also by creating a network in the medical (hospital) and social sectors (associations and clubs for older people).

Participants declared they heard about medico-social institutions “*on TV*” or “*on the radio*.” It is also a more ancient and popular system: “*And then, it's not new…we've always heard and seen this*.”

Regardless of the institution they lived in, the respondents had little knowledge about the other accommodation possibilities. Despite the variety of reasons and motivations for this choice, there was always an acceptance. In medico-social institutions, residents expressed it: “*nothing helps me, nothing bothers me*,” “*everything works for me, nothing works for me*,” “*you have to accept especially once you are alone, you cannot remain alone so it is better to accept what there is*.” They also said: “*I understood that I had to be somewhere*,” “*for family reasons, I agreed. Now it suits me*,” “*I am alive, I know I'm here*.”

There is a passive acceptance of the institutionalization by respondents who declared themselves: “*relatively well*” but suffering from a “*lack of compassion.”*

## Discussion

### Foster Care Families

#### Do Foster Care Families Constitute a Recommendable Alternative Housing for Older People?

The hosts of foster care families try to make themselves known on their own to develop their activity and have guests. This approach may be legitimized by taking into account the poor development of this system and the precarious status of the welcoming people (27). The legislation has not provided a possible use of return-to-work assistance in the event of loss of guests for any reasons. Their incomes depend only on their welcoming guests^3^.

The example of Martinique let us to think about the same reason which can explain the requests of foster care institution. Although the accommodation offer seems varied in Martinique, it remains insufficient in terms of capacity. In 2014, only 30 retirement homes, medico-social institutions, and residential homes, were available and allowed the accommodation of 49 inhabitants per 1,000 inhabitants aged 75 years and over (123 per 1,000 inhabitants in metropolitan France). There were also 140 places in Long-Term Care Units (USLD), 36 places in a day care centers, 56 in shelters, that is 1.8 places per 1,000 people aged 75 years or over (18.5 places per 1,000 inhabitants at national level). Additionally, there were 70 places in the Poles of Activities and Adapted Care, 130 places for Alzheimer's patients, 2,000 places in medical care, and 177 places in foster care families^4^.

This example of Martinican foster care families showed another problem that is the existence of a significant number of illegal foster care family institutions. The lower cost of this type of institutions compared to other types may be one explanation for their existence. For some hosts, it is possible that the motivation could be essentially financial. The former professional status (no professional activity, employees, workers…) of a number of hosts may suggest that this is a new professional activity, a professional reconversion, or a resumption of professional activity after a break-up? Looking back to the history of this system, it was somehow developed to put an end to the abuses in the care of older persons and to regularize their reception by individuals to improve their quality of life for a better aging (28). Centuries later, we are still facing unofficial and unregulated foster care families probably due to the limited number of places in medico-social institutions and the growing number of older people. It would be interesting to know more about the real reasons of the development of illegal institutions but also about the quality of life of older people living in these illegal accommodations in order to be able to detect any type of possible mistreatment (29).

Nevertheless, foster care family institution represents a relevant example of alternative housing (30), with a logic of proximity and family relay, which could present a solution for the reception and support of the older individuals, especially in peri-urban and rural zones, in order to allow them to keep living in a familiar environment. But also it can be used as a therapeutic reception solution for some older adults. These habitats are widely used as operating concepts in European countries and are successful because of their economic (less costly) and social benefits (favoring the autonomy of older adults). These notions have been evoked in previous studies (31–33).

They also represent a social innovation that will surely lead to positive transformations in the institutionalization system as it is the case for community housing in Quebec (34).

#### The Notion of a Foster Care Family: A Mixture of Two Concepts

The notion of a foster care family is a mixture of two concepts: reception/welcoming and family.

The welcoming family receives people at home, the quality of this welcome depends only on its hospitality, its way of being, and its personality. Both the guests and the hosts need some time for adaptation and acceptance of the other.

Indeed, the older person is a guest who can be considered by the host and their family and relatives (who can also play a part in this welcome) like an actual family member, a friend, a customer, or an intruder depending on the history of the foster care family and its projections (16).

Chauchard defined the concept of reception as a non-external act, but part of ourselves; we are biological and cerebral welcome structures. It can be said that human beings communicate and spontaneously create social bonds (35).

Bourdieu found that acceptance was influenced by the social environment of each person, which gave him a “habitus” (36). Chauchard and Bourdieu (but also Fischer, Dortier, and Greenwald) define the reception as an opening of the social bond, managed by the social and cultural organizations (37). In parallel of these definitions, the concept of reception for health professionals remains a separate concept, since reception becomes in this context a professional act that is learned and developed in favor of the quality of care.

Consequently, the hosts must be trained and somehow evaluated to ensure a good quality of reception and a better management of the older people benefiting from this system. Even if the hosts welcome older individuals in their own homes, they remain employees and guests remain their employers.

The term “foster care family” implies also the notion of family or even the composition of a family. The family is by definition a community of individuals united by kinship. For single-parent families, not living as a couple usually meant living with family members or friends who supported them and helped them to carry out their activity, and receiving relatives for long daily visits, which reflects the strong presence of the notion of extended family.

### Choosing a Foster Care Family or a Medico-Social Institution: Residents' Perceptions

The characteristics of the two groups seemed comparable from a medical and physiological point of view. However, the participants living in foster care families seemed to be more autonomous for the acts of daily life but also regarding decision making, as previously mentioned (38).

Institutionalization, whether in medico-social institutions or in foster care families, remained traumatic for older people, even if it seemed to be better experienced by people living in foster care families who considered that they are in transit and that the situation will change 1 day with a possible return at home. Somehow, they may think they are re-energizing themselves so that they can go back to living autonomously 1 day (30).

The confrontation with daily activities and schedules and the reconstruction of social links seemed to allow people to find a “normal” rhythm of life in foster care families. In other words, to be welcomed allows the acquisition or re-appropriation of new social skills. Most welcomed people have prospects of repossessing/returning to personal accommodation in the short to medium term (38). There is something good about it, since the foster care family is not experienced as a tipping point, it is not a trauma in itself for older adults. Perhaps that is why it is more bearable than medico-social institutions. In reality, this element, combined with an ordinary family life, seems to contribute to the transitional representation of the foster care family system. This situation could stimulate older individuals and this stimulation may allow them to continue managing their daily life, guide them in a thoughtful life choice: a return to home, a medical institutionalization. This system naturally appears as a possible extension of the family lifestyle of older people.

The medico-social institution is a professional structure whose staff members are paid to serve the residents without living with them nor sharing their daily life. Doesn't it go a little too far when they decide for and in place of residents, even if they mean well? In medico-social institutions, residents had the impression of being controlled, managed, and submitted to a lot of rules.

In foster care families, people felt freer, even if they seemed embarrassed to share with others certain rooms, such as toilets or bathroom, considered as intimate. The proximity with the other guests or with the hosts was not much appreciated by older people in foster care families. This caused a certain degree of discomfort compared to the institution, which was hardly tolerated by older people.

On the other hand, in the medico-social institution, a health professional organization, older individuals seemed to feel more “at home” since the institution does not belong to anyone and especially not to the professional caregivers who accompany them daily, the institution is in a way a structure to be shared between the residents, it is their “home.” People have their own personal space. But at the same time, they felt controlled and managed by care givers and the rules in place.

Although people living in foster care families appreciated the personal support provided, they expressed they are somehow constantly reminded that they are living in their care giver's home, not theirs. This can cause a feeling of insecurity. Ultimately the place of residence and not only the quality of care received can influence the quality of life of older people (39).

In response to the identified limits of the foster care family system, some countries have developed the model of the foster home centers for older people (40). In such a system, the welcoming care giver as well as the guest live at this home center. The welcoming care giver is employed by the center. Also in this type of accommodation, rooms are considered as “intimate” (e.g., sanitary) and completely private. Guests do not share their bathroom. To our knowledge, there is no study comparing the perceptions of residents in this type of institution compared to foster care families, which would however be interesting.

People living in medico-social institutions evoked the lack of nursing staff, which influenced the perceived quality of their support. Conversely, guests in foster care families seemed to appreciate the quality of their support thanks to the personalized support offered to them by their hosts who is always present and very familiar for them.

Older people with a slight-to-moderate loss of autonomy who live in medico-social institutions (that receive mainly dependent older people) felt that they are suffering from a kind of injustice because of the dependent residents who need caregiver support all the time.

The diversity of the offers of services, care, and accommodation is important for the support of older adults. Publicizing these offers is also important. Older people seemed to be unfamiliar with the other available possibilities and this ignorance not only denies them the opportunity to take advantage of another system that may be a better suit for them, but it also gave rise to a kind of judgment—and maybe fear?—that could have been avoided.

The present study seems relevant to provide important information about the experience of guests in foster care families and to compare it with the experience of older people living in medico-social institutions. As previously stated (41), it is indeed essential to take into account this expressed experience in order to improve the arrangements in terms of quality of services and responses to people's expectations (42) and, if necessary, to develop this system not only in Martinique but also in metropolitan France. In this way, public policy will be able to consider the social dimension of the foster care family to make older people more autonomous in their decisions and management of everyday life.

The strengths of the foster care family system comprise the personalized and human-scale support, the availability and benevolence of the hosts, the quality of the living environment, the quality of meals, the more active participation in daily life activities, the stimulation of autonomy, the wide choice of geographical situations, and the awakening of desires to do more activities and to have projects. This should encourage the conduct of more studies on the ways to improve this system while ensuring its sustainability and encouraging public support.

## Strengths and Limits

With the current lack of housing structures for older adults and their increasing number, it is important to study the available modes of support. By focusing on the foster care families, this project is helping to rethink the modalities of developing housing structures of the future and to propose ways to improve the management of seniors.

The present study provides an in-depth insight on the still unknown mode of reception (43) that is the foster care family system, and can therefore provide important information to determine whether foster care families should be developed, not only in the overseas regions but also in metropolitan France and maybe other countries as an alternative to home support and/or medico-social institutionalization as previously proposed (44).

These results could serve the health policies dedicated to the management of old age by reporting the words of the users themselves.

As for every qualitative study, the results are not generalizable to all seniors in these two types of accommodation. The purpose of the study was not to provide a comprehensive review of the situation of older individuals, but to analyze diverse situations of older people. We paid particular attention to integrating men and women, as well as participants with different socio-demographic characteristics (different ages, living environments, socio-economic levels, dependency levels, family structure, and region). Key professionals from medico-social institution were familiar with all residents and provided a list of participants, which may have induced an important selection bias. Nevertheless, people were very spontaneous in their interviews. The reformulation of the same questions at different times during the interview and in different ways confirmed the coherence, freedom, and spontaneity of their remarks.

It is not trivial to collect the word of older individuals concerning their place of life: the fear of conflicts or reprisals that can result from speaking out should be taken into account and analyzed. Thus, while the research topic is not sensitive, it can be disruptive for the respondents, and psychological support must be provided for such research. At least, it is necessary to mention to the staff members and to the hosts that this can constitute a sensitive topic.

Also, the recorded oral consent seemed to be a barrier to participation in the study. Some participants withdrew their consent to participate on the day of the interview because of this request for an oral consent. For the others, a certain discomfort was noted and disrupted the beginning of the interview. Consequently, the confidence with the participants was more difficult to reach for the first questions. We took up the reformulated questions, once the person was feeling more confident to ensure that he/she could tell us everything without holding back.

We are aware that among the health problems affecting older patients, Alzheimer's disease and Alzheimer-related diseases lead to situations that pose specific problems (45). However, problems of loss of autonomy related to severe cognitive disorders represent a very specific situation in terms of needs. We chose to focus this study on seniors with a loss of autonomy unrelated to Alzheimer's disease or Alzheimer-related diseases.

Another limitation is that we were unable to further explore data on unofficial foster care families, which undoubtedly represent a significant proportion of foster care families, as well as data on requests for placement in foster care families that have been refused and/or that are still waiting for an answer.

## Conclusion

The present study responds to the specific need of expanding the offer of accommodation for older people taking into account the cultural specificities of Martinique and the needs of older adults.

The results present the foster care family as an offer of accommodation with a human scale, a bit like at home, since it allows to create a small family unit of substitution for the guest and a life that appeared more flexible than the one in medico-social institutions. Nevertheless, a number of points need to be improved in order to better develop and support this offer, especially regarding the training of the hosts for a better reception, the size and arrangement of the accommodations so that the people keep their privacy, and the variety of animations and services to meet the needs of the guests but also those of the hosts.

In addition, this system presents an inclusive dynamic that must be analyzed in depth to ensure a complementary response more adaptable and adapted to older people and their families, but also to help public policies to continue improving the development of this system and its functioning, in terms of management of the reception, recruitment of the host, their training, and support as an intermediary habitat.

Further studies will be necessary to investigate, for example, the cost and cost-benefit ratio of the foster care families compared to medico-social institutions but also compared to home care in order to better guide public policies in the field of health economics. Besides, it offers the possibility of job creation for young adults and the revival of life in rural areas.

Further studies could be conducted to identify the profiles of older people likely to join a foster care family and those who will be better in a medico-social institution. This would make it possible to accompany older individuals and their families in the choice of the most suitable type of accommodation and would facilitate older people's adaptation to change while avoiding placement failures.

## Data Availability Statement

The original contributions presented in the study are included in the article/supplementary material, further inquiries can be directed to the corresponding author.

## Ethics Statement

The studies involving human participants were reviewed and approved by Ethics Institutional Review Board INSERM (Ref: IRB00003888, IORG0003254, and FWA00005831). The Ethics Committee waived the requirement of written informed consent for participation.

## Author Contributions

RC and SD: data curation and formal analysis. RC, SD, and AS: drafting and reviewing of the manuscript. SD: supervision. All authors contributed to the article and approved the submitted version.

## Conflict of Interest

The authors declare that the research was conducted in the absence of any commercial or financial relationships that could be construed as a potential conflict of interest.

## Publisher's Note

All claims expressed in this article are solely those of the authors and do not necessarily represent those of their affiliated organizations, or those of the publisher, the editors and the reviewers. Any product that may be evaluated in this article, or claim that may be made by its manufacturer, is not guaranteed or endorsed by the publisher.

## Footnotes

^1^https://www.insee.fr/fr/statistiques/2012690.

^2^https://www.collectivitedemartinique.mq/seniors/.

^3^https://solidarites-sante.gouv.fr/fichiers/bo/2013/13-07/ste_20130007_0000_0091.pdf.

^4^https://www.martinique.ars.sante.fr/la-semaine-bleue-7-jours-pour-parler-de-nos-aines.

## Appendix

**TABLE A1:** Research team.

**Sex**	**Credentials**	**Occupation**	**Experience and training**	**Location**
Female	PhD	Director of research	Professor Public health physician	Metropolitan France
Male		Fellow researcher	Geriatrician	
Female			Social psychologist	
Female	PhD-student	Junior research	Health science	Metropolitan France and Martinique
Male	PhD	Fellow research	Sociologist	Martinique
